# National and subnational HIV/AIDS coordination: are global health initiatives closing the gap between intent and practice?

**DOI:** 10.1186/1744-8603-6-3

**Published:** 2010-03-02

**Authors:** Neil Spicer, Julia Aleshkina, Regien Biesma, Ruairi Brugha, Carlos Caceres, Baltazar Chilundo, Ketevan Chkhatarashvili, Andrew Harmer, Pierre Miege, Gulgun Murzalieva, Phillimon Ndubani, Natia Rukhadze, Tetyana Semigina, Aisling Walsh, Gill Walt, Xiulan Zhang

**Affiliations:** 1 Department of Public Health and Policy, London School of Hygiene and Tropical Medicine, Keppel Street, London, WC1E 7HT, UK; 2 Health Policy Analysis Center, Togolak Moldo 1, Bishkek, 720045, Kyrgyz Republic; 3 Department of Epidemiology and Public Health, Royal College of Surgeons in Ireland, 123 St Stephens Green, Dublin 2, Ireland; 4 School of Public Health, Cayetano Heredia University, Avenue Armendariz 445, Lima 18, Peru; 5 Departamento Saude da Comunidade, Universidade Eduardo Mondlane, Praça 25 de Junho, Maputo, 257, Mozambique; 6 Curatio International Foundation, 37d Chavchavadze Avenue, Tbilisi, 0162, Georgia; 7 Beijing Normal University 19 Xin jie kou wai da jie, Beijing, 100875, China; 8 Institute of Economic and Social Research, University of Zambia, Lusaka, P.O. Box 32379, Zambia; 9 School of Social Work, Kyiv-Mohyla Academy, 2 Skovorody Vul, Kyiv, 04070, Ukraine

## Abstract

**Background:**

A coordinated response to HIV/AIDS remains one of the 'grand challenges' facing policymakers today. Global health initiatives (GHIs) have the potential both to facilitate and exacerbate coordination at the national and subnational level. Evidence of the effects of GHIs on coordination is beginning to emerge but has hitherto been limited to single-country studies and broad-brush reviews. To date, no study has provided a focused synthesis of the effects of GHIs on national and subnational health systems across multiple countries. To address this deficit, we review primary data from seven country studies on the effects of three GHIs on coordination of HIV/AIDS programmes: the Global Fund to Fight AIDS, Tuberculosis and Malaria, the President's Emergency Plan for AIDS Relief (PEPFAR), and the World Bank's HIV/AIDS programmes including the Multi-country AIDS Programme (MAP).

**Methods:**

In-depth interviews were conducted at national and subnational levels (179 and 218 respectively) in seven countries in Europe, Asia, Africa and South America, between 2006 and 2008. Studies explored the development and functioning of national and subnational HIV coordination structures, and the extent to which coordination efforts around HIV/AIDS are aligned with and strengthen country health systems.

**Results:**

Positive effects of GHIs included the creation of opportunities for multisectoral participation, greater political commitment and increased transparency among most partners. However, the quality of participation was often limited, and some GHIs bypassed coordination mechanisms, especially at the subnational level, weakening their effectiveness.

**Conclusions:**

The paper identifies residual national and subnational obstacles to effective coordination and optimal use of funds by focal GHIs, which these GHIs, other donors and country partners need to collectively address.

## Background

A coordinated response to HIV/AIDS remains one of the 'grand challenges' facing policy makers today [1]. As the number of global health actors continues to proliferate exponentially, one particular set of actors - global health initiatives (GHIs) - has the potential both to facilitate and exacerbate coordination. New actors bring new resources for health, increased flexibility and creativity, all of which require coordination. However, the diversity and complexity of relations amongst multiple actors - a hallmark of GHIs - may also weaken already fragile health systems, thereby undermining their efficiency, effectiveness and equity [2-5].

Whilst single country studies and broad-brush reviews are starting to reveal the complex relationship between GHIs and efforts to coordinate the HIV/AIDS response [6,7], synthesis of primary data from multiple countries is required to identify cross-country challenges and lessons learned. This study fills this knowledge gap by presenting a synthesis of primary data from seven country studies on the effects of the Global Fund to Fight AIDS, Tuberculosis and Malaria, the President's Emergency Plan for AIDS Relief (PEPFAR), and the World Bank's HIV/AIDS programmes including the Multi-country AIDS Programme (MAP).

At the global level consensus has emerged about the need to improve coordination of health and HIV-specific programmes [8-10]. Several initiatives have aimed at improving coordination (Table 1). In 2004, the UNAIDS 'Three Ones' principles called for one national AIDS coordinating body, while in 2005 both the Paris Declaration on Aid Effectiveness and the Global Task Team on Improving AIDS Coordination among Multilateral Institutions and International Donors (GTT) reported on how actors within the new global health architecture might better coordinate their activities. Buoyant with a new-found enthusiasm for coordination, a flurry of international activity in 2007 led to the establishment of the Global Implementation Support Team, the Global Campaign for the Health MDGs, and the International Health Partnership (IHP) - all calling for better coordination to achieve improved health outcomes.

**Table 1:** Global and country level initiatives, agreements and processes to promote coordination of health programmes

Global level	
2004	UN '3 Ones' Principles
2005	Paris Declaration on Aid Effectiveness
2005	Global Task Team on Improving AIDS Coordination among Multilateral Institutions and International Donors
2007	Global Implementation Support Team
2007	Global Campaign for the Health MDGs
2007	International Health Partnership (IHP) Global Compact

**Country level**	

1980s to date	National AIDS Commissions (NACs) or equivalent
1997	Sector Wide Approaches (SWAPs)
	Poverty Reduction Strategies
2001	Global Fund Country Coordination Mechanisms
2006	One-UN - 'Delivering as One'
2008/9	International Health Partnership (IHP) Country Compacts

At the country level the need for a coordinated HIV/AIDS response is also recognised as urgent, and numerous country-level programmes and reforms have been implemented with varying degrees of success (Table 1). Beginning in the late 1980s with the WHO's Global Programme on AIDS - the genesis of many current National AIDS Commissions (NAC) or their equivalents - efforts to coordinate were given a boost in 2002 with the introduction of the Global Fund's Country Coordinating Mechanism (CCM). Established to coordinate country-funding proposals and broaden cooperation and participation in decision-making, early experiences were mixed: some CCMs integrated with NACs, others developed complementary roles, and some were reported to be competing for the same resources [11,12]. In 2006 the UN's report *Delivering as One *added emphasis to the need for better country coordination by outlining a series of reforms to streamline the work of UN agencies operating at country level [13], and by 2009 Country Health Sector Teams were being developed through the IHP as a way to bring civil society and non-state actors into the coordination process [14].

The introduction of GHIs such as the Global Fund, PEPFAR and the World Bank's Multi-country AIDS Programme have important implications for these and other efforts at improving coordination of health programmes. While they have diverse governance arrangements - PEPFAR is a bilateral programme, the Global Fund is a public-private partnership and the World Bank is a multilateral agency - their common feature is the extent to which they have mobilised substantial resources for HIV/AIDS control in multiple countries. Brugha defines a GHI as: '*a blueprint for financing, resourcing, coordinating and/or implementing disease control across at least several countries in more than one region of the world*' [15]. Indeed these GHIs have mobilised unprecedented levels of funds for diseases such as HIV/AIDS, malaria and tuberculosis and engendered increased political attention and widened stakeholder engagement for disease control [6,16]. The Global Fund, for example, has rapidly scaled up its funding from less than 1% of total development assistance for health in 2002 to 8·3% in 2007, with total approved funding of 15.6*B *[17,18]. *PEPFARhascommittedover *3.8B in funds for HIV/AIDS programmes globally [19].

Concerns have been raised about how well GHI programmes are coordinated and aligned with health systems, and whether they have exaggerated problems of weak health systems in some cases. Some GHIs have required countries receiving funds to establish new coordination structures, as in the case of the Global Fund; others, such as PEPFAR, have operated relatively independently of national coordination systems. In the first, and to date only, systematic review of GHIs, the Global Fund was credited with expanding stakeholder engagement, notably civil society participation in CCMs, although in some countries governments dominated CCM decision making while sideling civil society and private sector actors [6]. While the Global Fund has since introduced tighter conditions stipulating the inclusion of these groups [20,21], CCMs have also been criticised for duplicating existing coordination structures, thereby adding to an already complex health governance architecture, and for failing to engender effective communication and trust between members [11,22-25]. PEPFAR has been criticised in particular for limited transparency, and a lack of willingness to coordinate with other donors [26,27], although the new Obama administration has pledged to revise PEPFAR's Country Operation Plans to ensure better coordination with country governments and donors [10].

Ten years have passed since the launch of the World Bank's Multi-country AIDS Programme, and almost five years since PEPFAR was launched. The Global Fund's Technical Evaluation Reference Group (TERG) has just completed its Five Year Evaluation, and findings from primary research about the effects of GHIs on health systems at national and subnational levels are beginning to be reported [27-39]. It is therefore an appropriate time to revisit and review the effects that GHIs providing large levels of funds to HIV/AIDS control are having on coordination efforts in-country. Most studies have been located in Africa and have focused on the national level. Now that GHIs are well established, knowledge is needed on their effects across more diverse country settings, and at subnational as well as national levels. This paper addresses some of these knowledge gaps by presenting a synthesis of empirical findings on the effects of three GHIs for HIV/AIDS across seven countries. While the results fill some gaps, what is striking from our findings is the paucity of data in some areas, in some countries, and for some - though not all - of the initiatives; but we argue that this is an important finding in its own right and that there remains an important need for ongoing studies on the effects of GHIs on country health systems as these initiatives mature.

Based on empirical evidence from country studies forming part of the *Global HIV/AIDS Initiatives Network *(GHIN) http://www.ghinet.org, this paper explores the effects on subnational and national coordination structures of three GHIs for HIV/AIDS control that collectively contribute more than two thirds of external funding for HIV/AIDS programmes [40]: the Global Fund, PEPFAR, and the HIV/AIDS programmes that form a part of the World Bank's Health Nutrition and Population (HNP) programme including the Multi-country AIDS Programme (MAP). Table 2 summarises the key features of each of these initiatives. The paper synthesises empirical qualitative data from seven country studies: two from Europe (Georgia and Ukraine); two from Africa (Mozambique and Zambia); two from Asia (China and Kyrgyzstan); and one from Latin America (Peru). These country studies were selected on the basis that: a) they were members of the GHIN network, and b) they had explored coordination as part of their study. Reports for the studies conducted in the seven countries are accessible at http://www.ghinet.org/[28-39]. Key reports are referenced fully in this article. The Peru research team has also published some of their findings at http://www.iessdeh.org/usuario/ftp/final%20ghin.pdf

**Table 2:** Focal GHIs for HIV/AIDS

	Global Fund	PEPFAR	World Bank MAP
Type of organisation	Public-private partnership	Bilateral donor	Multilateral agency

Date commenced	2002	2003	2000

Disease focus	HIV/AIDS, malaria, TB	HIV/AIDS	HIV/AIDS

Priorities	Set by country stakeholders presented through proposals	Priorities and targets set by US Congress	Based on national HIV/AIDS strategic plans

Management approach	Country Coordination Mechanisms and Local Fund Agents	National AIDS Council/secretariat	Coordinated through US embassies

Main recipients	Government, civil society, private for profit	Mainly US and international NGOs disburse to local NGO sub-recipients; small government grants	Government ministries, NGOs

Funds disbursed 2003 (2006)	$789.1 M ($1031.3 M)	$949.2 M ($2517.6 M)	$307.7 M ($36.1 M)

Source: adapted from Biesma et al 2009 [21]

The paper has the following objectives:

▪ To assess progress towards the Three Ones principle of creating one national AIDS coordination authority by mapping national and subnational coordination structures with a remit for HIV/AIDS across the seven countries;

▪ To identify how the above GHIs - where present - have affected national and subnational HIV/AIDS coordination structures including their creation, broad participation and effective functioning;

▪ To assess what has been achieved in terms of the functioning of national and subnational coordination structures and identify what problems remain.

Table 3 summarises GHI HIV/AIDS programmes in the seven countries together with selected indicators of HIV/AIDS; the table shows there is substantial diversity across these countries in terms of GHI country presence, epidemiological status (low level, concentrated or generalised epidemics) and amount of HIV/AIDS-related funding received.

**Table 3:** GHI HIV/AIDS programmes in seven case study countries

	HIV epidemic type (low, concentrated or generalised)	Number of people living with HIV (2000 and 2007)*	Adult HIV prevalence % (2000 and 2007)*	Global Fund HIV/AIDS grants (round, year and amount)**	PEPFAR allocation (year and amount)***	World Bank commitment (project title, duration and amount)
China	Concentrated	410,000 (2000) 700,000 (2007)	0.1 (2000) 0.1 (2007)	Round 3 (2004) $98 M Round 4 (2005) $64 M Round 5 (2006) $29 M Round 6 (2007) $6 M Round 8 (2009) $6 M	Not a PEPFAR focus country	N/A

Georgia	Low	< 200 (2000) 2,700 (2007)	0.1 (2000) 0.1 (2007)	Round 2 (2003) $12 M Round 6 (2007) $6 M	Not a PEPFAR focus country	N/A

Kyrgyzstan	Low	< 1000 (2000) 1,479 (2007)	0.1 (2000) 0.1 (2007)	Round 2 (2003) $17 M Round 7 (2008) $12 M	Not a PEPFAR focus country	Central Asian AIDS Program (2005-2010) (4 Central Asian countries) $25 M

Mozambique	Generalised	910,000 (2000) 1,500,000 (2007)	9.5 (2000) 12.5 (2007)	Round 2 (2004) $8 M Round 6 (2007) $23 M Round 8 (2009) $12 M	2004 - $37.5 M 2005 - $60.2 M 2006 - $94.4 M 2007 - $162 M 2008 - $228.6 M	N/A

Peru	Concentrated	53,000 (2000) 76,000 (2007)	0.4 (2000) 0.5 (2007)	Round 2 (2003) $22 M Round 5 (2006) $8 M Round 6 (2007) $24 M	Not a PEPFAR focus country	N/A

Ukraine	Concentrated	210,000 (2000) 440,000 (2007)	0.1 (2000) 0.1 (2007)	Round 1 (2004) $23 M Round 6 (2007) $14 M	Not a PEPFAR focus country	The World Bank program to fight HIV/AIDS and tuberculosis committed $77 M in 2003 but disbursements have been delayed

Zambia	Generalised	920,000 (2000) 1,100,000 (2007)	15.5 (2000) 15.2 (2007)	Round 1 (2003) $6 M Round 4 (2005) $115 M Round 8 (2009) $129 M	2004 - $81.7 M 2005 - $130.1 M 2006 - $149 M 2007 - $216 M 2008 - $269.2 M	Zambia National Response to HIV/AIDS (2003-08) $42 M

Source: * UNAIDS; **Global Fund website accessed 5/1/09 supplemented by country studies *** AVERT

The study embraces both deductive and inductive approaches to thematic analysis: we tested the importance of the key factors relating to the effective functioning of coordination structures identified in the literature in the seven country settings; additionally we identified and explored themes emerging from the country data. The literature to date defines the effective functioning of national coordination mechanisms including Global Fund CCMs in different ways [2,9,20,24,41-43].

• inclusive stakeholder representation across government departments;

• strong civil society engagement;

• appropriate level of membership;

• strong and effective leadership;

• authority and strong country ownership;

• alignment with other coordination structures;

• clear functions and mandates;

• clarity over structure, operating procedures and terms of reference;

• sufficient secretariat capacity; and

• effective communication between members.

**Figure 1:**
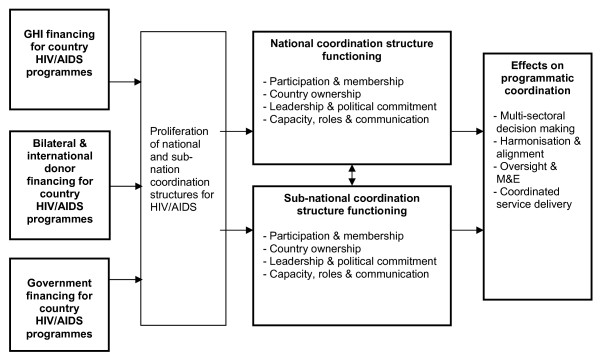
**Framework for assessing the effects of global HIV/AIDS initiatives on country coordination structures**.

Informed by these studies and the major issues grounded in the findings of the seven country studies we developed a health systems analytical framework (Figure 1) that captures a) GHIs and other financers of country HIV/AIDS programmes; b) aspects of the functioning of national and subnational coordination structures; c) and the effects of coordination structure functioning on programme coordination. Less data were available from these studies relating to c) the effects of coordination structures on programme delivery and health outcomes. While it has been widely accepted that improved coordination can lead to better efficiency, effectiveness, equity and sustainability of health and other programmes [2,44], this remains an area where further research is required.

## Methods

This paper draws on data generated from semi-structured interviews conducted by country teams with stakeholders from government agencies, civil society organisations (CSOs) and international partners at national and subnational levels between 2006 and 2008 in China (national and subnational n = 20; government n = 14, CSOs n = 4, international partners n = 2), Georgia (national n = 24; government n = 14, CSOs n = 3, international partners n = 7), Kyrgyzstan (national n = 36, subnational n = 60; government n = 41, CSOs n = 36, international partners n = 19), Mozambique (national n = 21; government n = 7, CSO n = 3, international n = 11), Peru (national n = 32; government n = 12, CSOs n = 12, international partners n = 8), Ukraine (national n = 30, subnational n = 105; government n = 37, CSOs n = 81, international partners n = 17) and Zambia (national n = 16, subnational n = 53; government n = 30, CSOs n = 35, international partners n = 4). Respondents, sampled purposively based on their involvement with GHI HIV/AIDS programmes, included government decision makers, international development partners, GHI programme implementers, HIV/AIDS service managers and other key informants in the HIV/AIDS-related field.

Based on these semi-structured interviews the studies aimed to elicit: a) information on the existence of national and subnational HIV/AIDS coordination structures, b) stakeholders' knowledge and experience of the effects of the focal GHIs on country health and HIV/AIDS systems including national and subnational coordination structures, c) key factors enabling and inhibiting the effective functioning of these coordination structures that remain despite (or resulting from) GHI-financed programmes, and d) key problems that inhibit the effective functioning of national and subnational coordination structures.

Each country team undertook systematic thematic analyses of their qualitative data, which were presented in country reports and supported by GHIN researchers at the London School of Hygiene and Tropical Medicine and the Royal College of Surgeons in Ireland. These findings were then drawn on to produce a comparative synthesis across the seven countries also utilising a thematic analysis approach [45]. The synthesis, which was led by the London and Dublin teams, adopted an investigator triangulation approach whereby multiple researchers contributed to analysing the findings in order to reduce personal bias and improve the internal validity of the synthesis. The synthesis involved:

1. Initial reading of all study reports and summaries of findings by the first analyst from the London team;

2. The London and Dublin teams met to agree a common analytical framework consisting of thematic headers;

3. Cross-country findings were systematically analysed by the first analyst with support from the Dublin team: findings were extracted from all study reports according to the common analytical framework and summaries of major findings tabulated;

4. Tables were reviewed by country teams to confirm the interpretation of each study's findings and input further study data where appropriate;

5. The paper was drafted by the first analyst and circulated to the London and Dublin teams for comment on its clarity on coherence;

6. The draft paper was reviewed by country teams to confirm accuracy of the representation of study findings and comment on its clarity on coherence, and the synthesis was agreed.

Ethical approval for the study complying with the Helsinki Declaration was granted by the London School of Hygiene and Tropical Medicine and by appropriate ethics committees in the countries where the studies took place where they exist.

## Results

### Proliferation of national and subnational HIV/AIDS coordination structures

A mapping of HIV/AIDS coordination structures at national and subnational levels shows that the architecture of HIV/AIDS governance in the seven study countries has increased in complexity. As Table 4 illustrates, in parallel to growing numbers of donors and initiatives financing HIV/AIDS programmes, new HIV/AIDS coordination structures have been introduced at national and subnational levels. NACs or their equivalent were in place in all seven countries before they received Global Fund HIV/AIDS grants. In some cases, multiple structures now exist at national and subnational levels either focussing on HIV/AIDS, or with HIV/AIDS a major remit. It appears that the seven countries have some way to go before realising the UNAIDS 'Three Ones' principle that calls for one multi-sectoral national body for HIV/AIDS coordination (Table 4).

**Table 4:** HIV/AIDS coordination structures in seven case study countries

Country	First national coordination structure with a remit for HIV/AIDS*	Year CCM was established	Current national coordination structures with a remit for HIV/AIDS*	Other national- level coordination structures with a remit for HIV/AIDS	Subnational coordination structures with a remit for HIV/AIDS
China	State Council Coordinating Mechanism for STIs and AIDS (1996)	2002	State Council AIDS Working Committee Office (SCAWCO) (2004)	-Most ministries have established HIV/AIDS coordination committees -The National Centre for AIDS/STD Prevention ontrol (NCAIDS), created in 1998 & integrated with Chinese CDC	-AIDS Working Committees -AIDS Prevention & Control Lead Groups

Georgia	Governmental Commission on HIV/AIDS/STI & other Socially Dangerous Diseases (1996)	2003	Country Coordination Mechanism (2003)	-National Centre for Diseases Control & Public Health -Prevention Task Force (PTF), est. under the USAID funded STI/HIV Prevention Project (UN agencies & national and international CSOs)	N/A

Kyrgyzstan	UN Thematic Group on HIV/AIDS (1996)	2001	Multisectoral Country Coordination Committee on Socially Significant Diseases & Especially Dangerous Diseases (2007)	-HIV/AIDS service CSOs Steering Group -Intersectoral Steering Group on Health Protection & Social Care in Penal Enforcement System - UN HIV/AIDS Theme Group	-Regional & municipal level HIV/AIDS coordination committees -Regional, municipal, district health coordination committees -CSO Working Group on Prevention of HIV/AIDS epidemic (Osh)

Mozambique	National STI/HIV/AIDS Control Programme within the Ministry of Health	2002	National AIDS Council (NAC) (2000)	-HIV/AIDS Partners Forum (link between NAC secretariat & donors) -Network of International CSOs working on Health & HIV/AIDS (NAIMA) MONASO: Network of national CSOs working on HIV/AIDS RENSIDA: National Network of PLWHA Associations CCM for Global Fund which meets mainly for project proposal review Health SWap: Sectoral Coordination Committee ('comite de coordenacao sectorial' (CCS), Joint Coordinating Committee ('sectoral co-ordination committee') (CCC), HIV/AIDS WGs/Taskforces	-Pre-partners forum (for HIV/AIDS) -Health Partners Group (for Health Sector)

Peru	Technical Commission for Notification & Registry	2002	Country Coordination Mechanism: National Multisectoral Coordination Commission on Health (2000)	Multisectoral National Coordination Committee on Health (Global Fund projects)	Multisectoral Regional Coordination Committees on Health

Ukraine	Governmental Commission on managing development and implementation of AIDS related countermeasures in Ukrainian SSR (1991)	2002	-Coordination Council on HIV/AIDS, TB & Drug Addiction (2007)	-UN Theme Group on HIV/AIDS -UN Joint Technical Team -National Council for HIV/AIDS & TB (2007) -Committee on HIV/AIDS & other Socially Dangerous Diseases (MoH) -Steering Group for World Bank Loan	-Regional & municipal level AIDS Coordination Councils -CSO Forum (Odesa) -Coordinating Groups of Sites (CGS) -District Councils on HIV/AIDS

Zambia	National HIV/AIDS Council (NAC) (created 2000; made legal by Parliament 2002)	2002	National HIV/AIDS Council (NAC) (created 2000; made legal by Parliament 2002)	- Cabinet Committee on HIV/AIDS -Thematic/Technical Working Groups - CCM - SWAp - ZANARA - CSO Networks: Zambia National AIDS Network (ZNAN); Churches Health Association of Zambia (CHAZ)	-District AIDS Task Forces (DATFs) & District AIDS Coordination Advisors (DACAs) -Provincial AIDS Task Forces (PATFs) & Provincial AIDS Coordination Advisors (PACA) -Provincial Development Coordinating Committee (PDCC) - District Development Coordinating Committee (DDCC) -District Health Management Team (DHMT) -Community AIDS Task Forces (CATF)

* Year structure was established

In China, Georgia, Kyrgyzstan, Peru, Ukraine and Zambia, Global Fund programmes stimulated the introduction of new HIV/AIDS coordination structures: in addition to national CCMs, subnational coordination structures have been created to coordinate local HIV/AIDS programmes [28-39]. In some countries, formal and informal structures and arrangements were initiated by civil society organisations (CSOs), governments and donors, although most were short-lived. Government and donor structures, for example, have consisted of loose coalitions of actors holding a one-off or time-limited series of meetings around particular issues/decisions. The HIV/AIDS architecture in Kyrgyzstan, which has a relatively low HIV prevalence (Table 4), provides ample illustration of this point. The country has formal coordination structures with a remit for HIV existing at four levels (national, regional, municipal and district-level), and structures in parallel to these including a national level NGO Steering Group; donor forums focusing on HIV/AIDS programme coordination; an Intersectoral Steering Group on Health Protection and Social Care in the Penal Enforcement System; and several local structures such as a Working Group in the Osh region which has the highest HIV prevalence in the country [28,29].

The studies in Mozambique, China and Ukraine in particular suggest that the multiplicity of parallel national and/or subnational coordination structures have challenged effective governance of HIV/AIDS programmes [34,35,37-39]. For example, specific challenges stemmed from individuals being members of multiple coordination structures; according to a respondent in Mozambique: '*[It is] ineffective to have multiple coordination structures: the same donor is a member of CCM, member of ICC and is also in the SWAp'*. Problems were reported in Ukraine, where multiple national and subnational HIV/AIDS structures exist within a complex, fragmented system of public administrative bodies inherited from the Soviet health system. The study revealed the multiple HIV/AIDS-related structures to have poorly-defined, delineated and overlapping objectives, functions and responsibilities that continue to embrace public sector working practices: their work was neither transparent, nor accountable, with no information about meetings and decisions taken being made public.

In some cases the transience of coordination structures has undermined their effectiveness. In the volatile political environments of Ukraine and Kyrgyzstan, HIV/AIDS coordination structures have been established (and abolished) several times, creating programmatic delays and confusion. Conversely, coordination efforts have benefited from relatively stable, albeit increasingly complex, coordination environments in Mozambique, Zambia and Peru. In Mozambique the CCM secretariat continued to exist as a separate entity, despite integration of the CCM into the SWAp Health Partners Group. In Zambia, the CCM has operated in parallel to the NAC and other national coordination structures [30,31,39].

Global Fund CCMs were diverse and integrated in different ways and to greater or lesser extents with other country structures, which demonstrates the Fund's evolution since the early years when CCMs were often stand-alone structures and seen as being imposed [22]. The CCM was the principal national HIV/AIDS coordination structure in Peru and Georgia; it formed a NAC sub-group (Ukraine, Kyrgyzstan); it was integrated within the Sectorwide Approach (SWAp) (Mozambique); it was a separate entity with NAC secretariat support (Zambia); and it was a separate entity but with substantial overlap of NAC membership (China) [28-39]. However the studies suggest that most CCMs continued not to perform the broad range of functions outlined in the Global Fund guidelines such as oversight and monitoring and evaluation: they primarily existed to agree and sign Global Fund proposals, and met infrequently. In Zambia, USAID and the World Bank sat on the CCM and PEPFAR provided technical assistance and financial support to the NAC [30,31].

### Participation and membership in national and subnational structures

A major goal of HIV/AIDS coordination structures is to promote multisectoral decision making, specifically to involve non-health government departments and nongovernmental actors. Earlier studies [11,46] and those reported here show that GHIs have widened stakeholder participation and engagement. World Bank supported HIV/AIDS programmes have increased multisectoral participation in Zambia, Kyrgyzstan and Mozambique, and World Bank country offices have participated in country structures in these countries, although not in Ukraine [28-31,34,35,39]. Global Fund CCMs in particular have improved multisectoral decision making: the majority of country studies suggest that the introduction of the CCM had improved participation in decision making across government departments (such as education, criminal justice and social care) and/or involvement of nongovernmental actors (Georgia, Peru, Kyrgyzstan, China and Ukraine) [28,29,32-38].

Nevertheless the studies suggest that despite these developments overall levels of participation and/or engagement of non-health government departments and nongovernmental actors in national and subnational coordination structures remained relatively modest. While no major groups were excluded from membership of national coordination structures in Mozambique and Zambia, in China, Kyrgyzstan, Georgia, Peru and Ukraine non-health government departments were either absent or had marginal engagement; indeed in those countries HIV/AIDS tended to be viewed as a Ministry of Health (MoH) responsibility reflecting the commonly held discourse that HIV/AIDS is a health rather than a broader social issue [28,29,32-39].

In the post-Soviet countries of Georgia, Kyrgyzstan and Ukraine, specialisation within the health system has inhibited interaction between different parts of the system, and between health and non-health departments [47]. Ukrainian and Kyrgyz respondents reported that this continued to undermine efforts to bridge divisions between AIDS, TB, drug services and STI services, as well as between government health and social care services receiving Global Fund HIV/AIDS grants [28,29,34,35]. Ukrainian respondents noted that government institutional cultures and management styles were resistant to change and there were few incentives to shift professional boundaries. Frequent changes among senior MoH managers in that country had undermined efforts to create partnerships across government departments and with international partners. In Ukraine and Kyrgyzstan high turnaround of individuals' membership in national and subnational councils, reflecting a volatile political context, was reported as disrupting their functioning [28,29,34,35].

Similarly poor coordination between government departments, between different levels of government and poor internal coordination/communication within some government agencies was also reported in China, although the establishment of the CCM was reported as improving government coordination around HIV/AIDS programmes. Additionally, in Kyrgyzstan the position of the national HIV/AIDS coordination structure had hindered attempts at multisectoral decision-making: the structure was relocated from Presidential to MoH level in 2008 [28,29]. As a respondent suggested, this impacted on multisectoral engagement in HIV/AIDS decision- making:

*We tried really hard for a long time to make HIV/AIDS problem to be recognised as a social problem in our country. However, if the Secretariat is now by the Ministry of Health, it means that HIV/AIDS became the health problem again*.

The studies suggest that all three GHIs have created opportunities for CSO involvement in HIV/AIDS programmes through funding their activities, or insisting on their inclusion in CCMs (Global Fund). The Mozambique study reveals that the integration of the CCM within the SWAp increased national level engagement of CSOs and the private sector. Similarly the research in Zambia found that CSOs have begun to play a significant role in district coordination structures, and the World Bank, through the Zambia National Response to HIV/AIDS Project (ZANARA), supported community responses to HIV/AIDS by financing community based organisations, which also participate in District AIDS Task Forces and Community AIDS Task Forces [30,31]. However, PEPFAR-funded implementers frequently remained outside subnational structures and worked directly with NGOs. Respondents believed that this led to inefficient use of resources and duplication of services. Other studies have also found significant progress in expanding the representation of CSOs on NACs and Global Fund CCMs (for which the NAC provides secretariat support) [41].

In Georgia the CCM membership was described as too large to be manageable. Lead ministries had more than one representative, while other ministries and NGOs were poorly represented: the private sector, religious organisations and education were absent. In order to address this problem the number of CCM members was decreased from 46 to 30 and a rotation principle introduced to manage civil society representation whereby NGOs would elect their representative annually, with two NGOs acting as permanent CCM members. This approach also ameliorated some of the problems of conflicts of interest among NGOs receiving Global Fund grants [36].

However, in common with previous studies and reviews [6,22,48], CSOs and vulnerable groups continued to play relatively limited roles in some coordination structures even where they were formally members. They were often absent from meetings and when present their contributions to discussions were limited compared to more major players such as the MoH (China, Kyrgyzstan, Ukraine, Zambia and Peru) [28-35,37,38]. Multiple barriers to effective participation were revealed in the GHIN studies, including:

• Competition for scarce resources at national and subnational level that created distrust between country organisations (including government departments and nongovernmental implementers) and hence a substantial disincentive to meaningful engagement in coordination structures (Peru, Kyrgyzstan, Zambia and Ukraine);

• Limited experience among most CSOs of engaging in strategic or political decision making;

• Limited financial resources and time to commit to meetings including costs of travelling, and no financial incentives such as per diems and honoraria to encourage attendance (Kyrgyzstan and Ukraine);

• Insufficient time to contribute to proposals with tight submission deadlines (Peru);

• Government officials at national and subnational level selected CSOs to participate in coordination structures thereby excluding others (China) [28-35,37,38].

### Country ownership of national and subnational coordination

Unless coordination structures have authority and are seen to be under country ownership, any decisions they make may be ignored potentially leading to poor alignment of GHI and donor programmes with government priorities. The studies explored the extent to which donors were accountable to country coordination bodies and the strength of leadership and political commitment to HIV/AIDS programmes. In Peru and China the studies showed that NACs were able to make decisions and to allocate resources to HIV/AIDS programmes. By comparison national and particularly subnational structures in Zambia, Mozambique, Ukraine and Kyrgyzstan had limited authority to make decisions or allocate resources to HIV/AIDS programmes [28-31,34,35,39].

An important reason for this was that major donors for HIV/AIDS programmes including PEPFAR continued to set priorities outside national and subnational structures; and their participation in such structures was seen as a formality. Donor interests continued to undermine country ownership and make coordinating multiple aid programmes difficult for countries [2,49]. The Kyrgyz, Ukrainian and Zambian studies reported that donors including GHIs did not fully engage in coordination structures so as to maintain institutional visibility and attribute impacts to the activities they had financed [28-31,34,35]. This was reflected in donors' unwillingness to relinquish control of funds to national or subnational coordination structures and to share information with other partners. A respondent in Zambia explained:

*... most people, when you ask them where they were working, they will tell you that they are working for the [donor] funded project. It's never a Zambian project. So I would like to see a situation where it is... The logo on the vehicle should just say: the Zambian national response to HIV/AIDS and not tell us where the money is coming from*.

In Zambia and Mozambique the studies found that national coordination structures could not hold the myriad of donors and implementers to account for the effectiveness of their programmes, especially those CSOs that received funding through other channels. PEPFAR and the World Bank participated in NACs in those countries, but PEPFAR recipients in Zambia had limited engagement in subnational coordination structures. Limited decision making and resource allocation powers have been particularly acute within subnational structures, which in practice worked as implementers of local programme determined at the national level rather than as coordination bodies. Donors frequently bypassed such structures. In Zambia government subnational coordination structures, the District AIDS Task Forces, have had a technical/coordination role rather than decision making or resource allocation powers: respondents observed that there was no obligation for GHI-funded NGOs to report to District AIDS Task Forces; they frequently worked to their own priorities and did not participate in them. As a consequence these structures have had very limited control over donor activities and those of international NGOs, and often had minimal information on their activities including how PEPFAR funds were being spent in their districts. Some informants suggested that donor funds were being allocated to programmes which did not coincide with district priorities, leading to service duplication [30,31]. One respondent explained:

*One of the challenges when a donor moves into the district, you just see a donor is working there. All they will say is we have been to the Ministry of Health or Education, we got permission and we are working here*...

The positioning of coordination structures within the wider public administration system has important implications for levels of country ownership and the authority a structure can exercise. An important reason for positioning NACs under the Presidential Office in some African countries has been to give the structures political legitimacy and demonstrate political commitment [42]. In Kyrgyzstan the national coordination structure lost the authority that it had prior to 2008, when it was directly responsible to the President's Office. Subsequently, the secretariat, which reported to the MoH, was perceived as having little authority, acting as little more than 'a petitioner' of information from member agencies. Subnational coordination structures in Kyrgyzstan also lacked authority since NGOs were mainly accountable to donors on whom they were highly dependent. They were not financed through government budgets and/or coordination structures, making them more aligned to donor requirements. In practice NGOs were not obliged to report to these structures, thereby undermining the ability of the structures to coordinate local programmes [28,29].

Similarly in Ukraine the NAC has had an advisory rather than a decision-making function and met only to agree Global Fund proposals, at which point it was labelled a CCM. Subnational structures had very limited decision making power and minimal influence over local budgets for HIV/AIDS programmes [34,35]. A respondent suggested that the national structure had:

*... the status of an advisory institution; that is it doesn't make any decisions... the Coordination Council should help coordination. And this is what they don't do. They meet, review issues, make decisions which are often not implemented*.

In Zambia, Peru, Ukraine and Kyrgyzstan subnational HIV/AIDS coordination structures were seen as particularly weak and as reinforcing centralised decision-making. In Peru respondents reflected on the limited input from subnational stakeholders in preparing Global Fund grant proposals since the need to draft the proposal rapidly made broad participation and consultation from subnational stakeholders impossible. In Zambia there were mixed views from respondents about whether in practice planning was top-down (from the NAC to the district level) or bottom-up. According to the Zambian National HIV/AIDS Strategic Framework, it was yet to be established how the NAC should communicate with lower level structures and the flow of information to NAC from structures at lower levels was not yet clearly outlined. In Ukraine respondents saw the creation of subnational coordination structures as imposed from the national level and/or international donors, and that their decision-making powers to shape programmes and allocating resources were limited. Regional HIV/AIDS coordination committees were a requirement under the terms of the Global Fund grant, although the grant was not used to fund their establishment or recurrent costs [28-35]. A respondent explained:

*... as a whole this system is still bureaucratic, vertical [structures] are created... those coordination councils are created down to the bottom, but everything is like it's used to be. Meetings, conferences, happy reports, everything is done, but the epidemic is spreading*...

### Leadership and political commitment

Leadership invested by key members of coordination bodies and commitment of high-level government leaders are important factors in controlling HIV/AIDS epidemics in countries [50]. Although a number of early studies suggested NACs lacked consistent leadership [51-53], our findings show improvements and good practice in other settings. In China government leadership of the NAC was strong. In some districts, for example Duyun and Guizhou, local government had a strong oversight role and had strengthened coordination structures leading to improved local programmes, although in other districts leadership was weak.

The Georgian CCM benefited from the strong leadership of the First Lady, resulting in improved attendance, coordination between ministries, and expedited decision-making. Kyrgyzstan reported committed leadership in some regional coordination structures, although in practice leadership was vulnerable to rapid turnover of members. Strong leadership was also observed in Mozambique and Zambia. Only in Peru was it reported that weak leadership had undermined the NAC's performance [28-33,36,39].

Political commitment is illustrated in different ways. In Peru a 'Declaration of political commitment to HIV/AIDS' raised the profile of the disease, and invoked greater multisectoral commitment than previously. However, no formal policy on coordination or partnership existed, which limited progress. In China the government obligated ministries and local government departments to establish coordination structures and engage with issues of HIV/AIDS. Commitment to coordinated working was found in the Zambian National HIV/AIDS Strategic Framework 2006 - 2010 and the Joint Assistance Strategy; and in Kyrgyzstan a number of government policies explicitly call for multisectoral and CSO engagement in HIV/AIDS control [30-33,37,38].

The Ukrainian study revealed variable levels of commitment from local government administrations to HIV/AIDS, which had impaired the effectiveness of coordination structures [34,35]. However, the introduction of HIV/AIDS coordinators in some regions financed by Global Fund HIV/AIDS grants strengthened leadership, improved local commitment and facilitated more effective coordination. Similar posts were created in some districts of Zambia with United Nations Development Program funding, although it was difficult for them to operate due to erratic funding from the NAC for DATFs which they coordinate [30,31].

### Capacity, roles and communication

Low capacity of secretariats in terms of experience, salaries and equipment, and limited clarity about roles among coordination structure members can undermine the working of these bodies [20]. Putzel notes that in some African countries NACs have been ill-informed and poorly motivated, and this was borne out in some of the studies reported here [24]. In Zambia, Ukraine and Kyrgyzstan, international donors did not allocate funds specifically for coordination structures, and these countries experienced problems stemming from the limited capacity of their secretariats. In Kyrgyzstan, respondents noted several problems, including changes in the Country Multi-Sectoral Coordination Committee (the national structure with a remit for HIV/AIDS) that led to secretariat staff being replaced. This meant that new secretariat staff were not sufficiently trained and were under resourced in terms of premises, equipment, and access to the internet, office supplies and salaries [28-31,34,35].

In Kyrgyzstan, China and Ukraine, respondents reported that Global Fund funding had engendered better communication and transparency between partners and improved clarity of roles and responsibilities [28,29,34,35,37,38]. For example in China the Global Fund programme had promoted greater attention on effective communication and cooperation between local government departments through regular meetings and jointly run programmes under the leadership of local CDCs. Ukrainian respondents saw the creation of the CCM as offering a model of cooperation and transparency between governmental and nongovernmental organisations that was starting to be taken up more broadly. According to one respondent:

*The Global Fund helped the coordination council understand more clearly and accept international procedures, the procedures of openness, open decision- making, transparency, because the Global Fund influenced indirectly the composition of the National council*.

A lack of clarity over division of roles and responsibilities among coordination structure members was reported in a number of countries (China, Kyrgyzstan, Peru and Zambia). Poorly defined roles among NAC members in Peru delayed the implementation of the Global Fund grant, and in Zambia roles and responsibilities were ill-defined between the NAC, MoH, other ministries and CSOs, and between various subnational structures and actors [30,31]. In Kyrgyzstan agreed working procedures were lacking, and the restructuring of the country HIV/AIDS coordination structure to encompass 'socially dangerous diseases' (infectious diseases in humans and livestock) resulted in a loss of clarity over the structure's role and focus [28,29]. Illustrating this issue a Kyrgyz respondent commented on the lack of focus of the current structure:

The time of people, who are members of Country Multisectoral Coordination Committee is very 'expensive'. And when I see that the agenda includes discussion of issues related to animal health, and only one of the three issues is related to HIV and my work, I ask myself, do I really need to go to this meeting?

Only in Mozambique did the country study suggest that roles were clearly defined among members of national coordination structures, in particular after the SWAp structure was streamlined in 2007.

Evidence of limited information flows within and between coordination structures was a key finding in most of the countries, which undermined meaningful exchange between members. While there had been considerable improvements in transparency between subnational actors in Zambia, PEPFAR and NGOs funded by the initiative were unwilling to share information with District AIDS Task Forces, which undermined their authority. However, those CSOs that did participate in these Task Forces were credited with improving communication sharing at district level [30,31].

In Kyrgyzstan limited formal coordination existed at all levels, and in the Ukraine working practices were neither transparent nor accountable. While Kyrgyz stakeholders reported that some local coordination councils fostered improvements in informal information exchange, limited *formal *communication continued to exist at all levels, and there remained a lack of transparency among actors [28,29]. Speaking about the national coordination structure a respondent said:

*At Country Multisectoral Coordination Committee meetings we cannot possibly get detailed information concerning... what and how much funds have been spent. We asked for this information so many times already, but all our attempts failed. We just receive general reports back*...

Competition for scarce resources at national and subnational level in Peru, Kyrgyzstan, Zambia, Mozambique and Ukraine was reported as creating distrust between country actors. Nevertheless World Bank HIV/AIDS programmes in Zambia and Mozambique have provided capacity support to the NAC secretariats, and are credited with improving transparency and communications [28-35,39].

## Discussion

### Towards programmatic coordination?

The empirical evidence collected in these seven countries provides a kaleidoscope of experience and throws light on country systems and their responses to GHIs. There is huge contextual and historical diversity within and between countries, although what is striking about these findings is that countries with very different contexts shared similar experiences of problematic coordination and the effects of GHIs: findings in Zambia and Mozambique, with generalised HIV/AIDS epidemics and high levels of HIV/AIDS financing, were similar to those in the low and concentrated HIV/AIDS epidemic countries of Europe, Asia and Latin America. In common across the seven countries is the finding that the GHIs - in particular the Global Fund - have had many positive effects on national level coordination. The evidence is that substantial new funding for HIV/AIDS control, for which GHIs can take most of the credit, has created opportunities for multisectoral participation, promoted greater political commitment and increased transparency among most partners.

However, refractory problems reported in earlier studies [11,46] still existed in 2006-08. These included the complexity of aid architecture relating to HIV/AIDS programmes in all seven countries, even in the low and concentrated epidemic countries where levels of financing are substantially lower than in the generalised epidemic African countries: such a trend is clearly at odds with the Three Ones principle of establishing a single national AIDS coordination authority. Donor fashions and attachment to their own procedures, especially in monitoring and evaluation, and patchy accountability to country-led structures were also substantial problems. Donor practices continued to undermine consistent alignment with country priorities and processes and lacked harmonisation among themselves [54,55] despite many internal and external evaluations [11,22-24,46,54]. Moreover, donor-generated competition for resources leading to reluctance to share information impaired local oversight of programmes and delivery systems thereby undermining monitoring and evaluation and the application of evidence at national and subnational levels to improve programme delivery. Systemic weaknesses in countries' national and subnational coordination structures were undermining the goals of the GHIs.

The new knowledge that this cross-country synthesis has begun to generate is that it is at the ***subnational level ***that the biggest gap between intent and practice was found in 2006-08. This is a particularly problematic trend. It contradicts the growing emphasis on decentralised health sector decision-making that is seen as strengthening the powers of local-level actors in the formulation and implementation of policies and programmes, thereby increasing responsiveness to local needs [56]. The studies revealed that early and refractory problems at the national level - including coordination structure proliferation, lack of ownership and capacity, and poor communication - were being replicated at subnational levels. The studies in Zambia, Peru, Ukraine and Kyrgyzstan revealed that problems of limited decision-making and resource allocation powers were particularly acute within subnational structures [28-35]. Indeed weak subnational coordination was seen as reinforcing centralised decision-making. In practice they functioned as overseers of government-funded and/or Global Fund programmes that were designed at the national level; or of donor programmes, including PEPFAR, which sidelined them. These findings accord with previous evaluations of the Global Fund in Ethiopia and Benin where programmatic planning was initially top-down and conflicted with national policies and processes for decentralisation [57,58].

Many PEPFAR recipient organisations in Zambia did not participate in subnational coordination structures including District AIDS Task Forces, which consequently had little control over these programmes [30,31]. Subnational structures also lacked information on programmes run by other donors or international CSOs. Similarly, subnational coordination structures in Kyrgyzstan lacked authority primarily because CSOs working in HIV/AIDS were not financed by - and were therefore not accountable to - these structures [28,29]. CSOs often did not inform them about their work, undermining their ability to coordinate activities, because they saw themselves as accountable to the Global Fund Principal Recipient and other donors on whom they depended for funding. This made them more aligned to donor priorities than to those set by national or local government. In Ukraine respondents saw the creation of subnational coordination structures as imposed from the national level and as having limited authority [34,35].

Given these tensions, it is not surprising that coordinated HIV/AIDS programmes remain a distant goal for many countries. These studies suggest that poorly functioning coordination structures undermine programmatic coordination, including weak multisectoral decision making, poor levels of oversight and monitoring and evaluation, poor alignment between GHI and donor programmes and national and subnational level priorities, and implementation problems including delays and confusion, inefficient use of resources and duplication of services. The Global Fund, PEPFAR and the World Bank have made an immense contribution to reducing the burden of HIV/AIDS, especially in sub-Saharan Africa. A clear lesson from these country studies is that GHIs and other donors, as well as national governments, now need to acknowledge and address the residual problems in national level coordination and focus more attention and resources on strengthening subnational coordination, if the gap between intent and practice is to be narrowed.

There are a number of limitations of the studies drawn on as part of this analysis. Firstly, much of the data focus on the Global Fund, which is present in all seven countries and has transparent processes, which made data collection easier. Less information on World Bank HIV/AIDS programmes (China, Kyrgyzstan, Ukraine and Zambia) and PEPFAR (Mozambique and Zambia) reflects difficulties in accessing data, and/or patchy engagement by these GHIs in coordination structures in some countries. It is therefore more difficult to generalise about the effects of the World Bank HIV/AIDS programmes and PEPFAR than about the Global Fund. Secondly, less data are available on subnational coordination than national coordination since subnational interviews were not part of the study design in Peru, Mozambique, and Georgia, although national interviewees commented on subnational coordination in Peru. Thirdly, the findings are based on qualitative interview data. Much less documentary evidence was available to corroborate these data, although country teams endeavoured to triangulate interview data to boost the validity of findings. Finally, while studies explored common themes, there was some heterogeneity across the studies in terms of the precise questions interviewees were asked.

## Conclusions

The evidence suggests that all seven countries are far from realising the UNAIDS 'Three Ones' principle of one multi-sectoral national body for HIV/AIDS coordination. National as well as subnational coordination structures with a remit for HIV/AIDS are proliferating, and in some countries the multiplicity of parallel coordination structures has challenged the effective governance of HIV/AIDS programmes.

GHIs are having some positive effects on HIV/AIDS coordination structures, as well as a number of negative effects: while much has been achieved, particularly at national level, many serious problems remain. For instance GHIs have widened stakeholder participation in coordination structures, although engagement from non-health government departments and civil society remains modest. Country ownership of national and subnational coordination is undermined by the weak decision making authority of many coordination structures and limited or perfunctory engagement among GHIs and other donors, particularly at the subnational level. There is evidence that strong leadership within coordination structures and broad political commitment to coordinated approaches to HIV/AIDS programmes have been improving, although weak secretariat capacity, poorly defined roles and responsibilities among members of coordination structures, limited transparency and communications and competition for scarce resources remain persistent problems undermining effective coordination.

Despite the many problems of coordination revealed above, there are several practical lessons stemming from the studies that decision-makers in these and other countries might bear in mind when seeking to strengthen the functioning of national and subnational coordination structures. These include the need to augment the capacity of secretariats of both national and subnational coordination structures through financial and technical support, and to carefully consider how best to position a national coordination structure within the public administration system in order to boost its authority and ability to promote multisectoral working. Financial support for CSOs could promote their effective participation in national and subnational coordination structures by enabling them to attend meetings. Other forms of support for CSOs might also be appropriate such as providing training in strategic or political decision making so that they are better able to engage in coordination meetings and more fully contribute to discussions. Clarity about roles and functions was often missing from the examples presented above reinforcing the need to develop and agree terms of reference for the objectives, functions and working practices of national and subnational coordination structures, and to define the roles and responsibilities of individual members.

Several knowledge gaps remain: follow-up research would be especially valuable in helping to better understand how the functioning of coordination structures plays out in the effective coordination of health interventions at the programmatic level, including coordinated service delivery. In particular, further research could help understand the functioning of subnational coordination structures and their effects on programmatic coordination since the evidence at subnational level from these and other studies remains weaker than that at national level. Moreover, at present most evidence is based on qualitative data collection: it will be important to build a stronger evidence base derived from both qualitative, as well as robust quantitative, measures to demonstrate the effectiveness of coordination structures and their effects on programmatic coordination.

## Competing interests

The authors declare that they have no competing interests.

## Authors' contributions

NS led on drafting this article. NS, JA, RB, RB, CC, BC, KC, PM, GM, PN, NR, TS, AW, GW and XZ all participated in the conception, design and execution of the study and analysis and interpretation of data. AH contributed to the analysis and interpretation of data. All authors participated in manuscript writing and have read and approved the final manuscript.
